# The impact of physical exercises with elements of dance movement therapy on the upper limb grip strength and functional performance of elderly wheelchair users living in nursing homes – a randomized control trial

**DOI:** 10.1186/s12877-021-02368-7

**Published:** 2021-07-12

**Authors:** Natalia Wołoszyn, Agnieszka Wiśniowska-Szurlej, Joanna Grzegorczyk, Andrzej Kwolek

**Affiliations:** 1 Institute of Health Sciences, Medical College of Rzeszow University, Warzywna 1A Street, 35-310 Rzeszów, Poland; 2 Institute of Medicine, Medical College of Rzeszow University, Warzywna 1A Street, 35-310 Rzeszów, Poland

**Keywords:** Aging, Physical health, Wheelchair user, Nursing homes

## Abstract

**Introduction:**

Over the last few decades, the quality of care and the quality of life of nursing home (NH) residents have significantly improved, but insufficient physical activity and social involvement still represent substantial challenges in modern nursing facilities. The main aim of this research was to assess the influence of physical exercises with dance movement therapy (DMT) elements on strength and other fitness components of the upper limbs and the overall functional performance of NH residents in wheelchairs compared to standard exercise programmes and usual care.

**Method:**

The study participants were persons aged 68–85 who lived in NH and used manual wheelchairs as a primary means of mobility. Individuals meeting the inclusion criteria were assigned to one of the three groups: Group 1, basic exercise/BE group (*n* = 55); group 2, physical exercises with elements of dance movement therapy/PED group (*n* = 55); and group 3, control group, usual care/CO group (*n* = 55). The intervention for both exercising groups consisted of a 30-min session, two times a week, for 12 weeks in total. Outcome assessments were performed at baseline, 12 weeks after baseline (immediately after the intervention) and 24 weeks after baseline (12 weeks after the intervention). The main outcome was observed for hand grip strength (HGS), while secondary outcomes for box and block test (BBT), arm curl test (ACT), back scratch test (BS), chair sit-and-reach (CSR), peak expiratory flow (PEF), Barthel Index (BI), Berg Balance Scale (BBS) and the range of motion of the shoulder.

**Results:**

Prior to the start of the exercise programmes, all the tested groups were homogeneous. After 12 weeks the PED group presented higher statistically significant scores in HGS_L_, BBT, ACT, BS, CSR, BI, BBS: *p* < 0.001 and HGS_R_: *p* = 0.01, compared to the BE group. After 24 weeks from the beginning of the intervention the comparison between the PED group and the BE group showed statistically significant differences (*p* < 0.001) in favour of PED group in almost all areas: HGS_R_, HGS_L_, BBT: ACT, PEF, BS, CSR, BI. After 12 and 24 weeks both intervention groups performed better than the CO in all measures except for Katz ADL and shoulder extension.

**Conclusion:**

Twelve weeks of physical exercises had beneficial effects on the strength and fitness of the upper limbs and overall functional performance in both exercise groups. This study demonstrated that group performing physical exercises with elements of DMT obtained statistically better scores in the majority of analysed domains than other groups.

**Trial registration:**

The study was registered in the Sri Lanka Clinical Trials Registry (Registration Number - SLCTR/2018/014 - Date of Registration 16/05/2018. Accessed on https://slctr.lk/trials/1045).

## Introduction

Demographic projections for the coming years clearly indicate a steady increase in the population of elderly people; an increased demand for long-term care is also expected [1]. The ageing of society has prompted the development of social policies that focus on the elderly and involve the establishment of mechanisms to meet the needs of seniors [2].

In many countries, numerous nursing home (NH) residents use wheelchairs as a means of mobility [3]. Wheelchairs are an assistive device and are a means of mobility for NH residents who experience mobility difficulties due to reduced muscular strength, fear of falling, visual impairment, or pain. The ability to move around using a wheelchair provides a certain amount of independence in everyday life and thus is a factor influencing the quality of life [4].

Adroit use of wheelchairs is conditioned by complex interactions between predictors, such as strength and flexibility of the upper body, grip strength, overall fitness of the upper limbs and mental fitness [5].

Many NH residents find it difficult to unlock their wheelchair to move around, which is the result of poor muscle strength and low joint mobility [4]. According to the research conducted, performing moderate-intensity physical exercise, even at an older age, contributes to an increase in strength and muscle mass. Additionally, exercise reduces the difficulty of performing daily tasks, increases energy expenditure, and affects the composition of the body (by replacing body fat with muscle mass) [6, 7]. Upper limb fitness, including manual dexterity, also plays an important role in everyday life [8].

On a hierarchical structure of disabilities, seniors are primarily faced with difficulties when performing activities requiring balance, agility, and strength*.* Consequently, skills requiring manual dexterity are lost. The longest lasting skills are the activities associated with washing one’s face and hands or eating on one’s own [9, 10]. These studies suggest a complex, multi-factorial decline in functional performance of the elderly and it is therefore important to include strategies aiming at improving both upper and lower limb fitness, as this is a crucial aspect of maintaining independence in a growing population of elderly people [11, 12].

Over the last few decades, the quality of care and the quality of life of NH residents have improved and become more effective, but studies show that most NH residents are largely inactive and spend most of their time lying idle or sitting alone [13, 14]. The lack of appropriate physical activity deteriorates physical fitness, which may eventually lead to a further increase in functional limitations and disability. Moreover, according to the World Health Organization, deteriorating physical fitness is the greatest health risk factor for elderly people [15].

Many authors acknowledge that physical activity has a positive impact on the functional performance of elderly individuals [16, 17]. In a systematic review of the literature, Anthony et al. demonstrated a positive impact of exercises performed by elderly individuals in sitting positions within three areas: functional performance, cardiopulmonary capacity, and mental health [18]. Furthermore, in their recent systematic review, Cordes et al. demonstrated that chair-based exercise interventions have a positive effect on the physical and cognitive functions and psychosocial well-being of NH residents [19].

The introduction of dance elements into exercises combines the benefits of physical activity with psychosocial benefits [20]. Elements of DMT, such as music and movement exercises or movement improvisations, effectively improve balance, functional performance, motivation and social integration. Group exercises with dance elements relieve the feeling of fear and social isolation [21, 22].

Senior citizens in wheelchairs are a heterogeneous group with different illnesses, disabilities, and preferences. The current physical activity models do not consider individual deficits and opportunities [23]. The existing studies do not provide evidence-based guidelines on exercise for NH residents using wheelchairs that would strengthen their health resources and prevent or delay the loss of physical function [24].

A review of the global literature, particularly focused on randomized control studies, allowed for us to identify a small number of reports on the impact of physical activities on the functional performance of elderly people, wheelchair users, and NH residents [19, 25]. For example, Chen et al. showed that wheelchair-bound elastic band exercises, significantly improved the functional performance of older adults with dementia on wheelchairs [26]. Thurm et al. reported that a 10-week multimodal movement intervention performed in the sitting position can slow cognitive deterioration of NH residents with dementia and physical frailty [27]. Eyigor et al. showed that folk dance improves physical fitness and some domains in the quality of life scale among healthy women [28]. However, Murrock and Graor indicated that a 12-week dance intervention may be an effective adjunct therapy to diminish depression and disability and to improve physical functioning among underserved adults [29].

Chin et al. demonstrated in their systemic review that the impact of physical exercises taken by elderly persons on their fitness is not unequivocal. Although physical training improves functional performance, the authors suggest that further high-quality studies are required to determine the most effective exercise programme [30]. A taskforce, under the auspices of The International Association of Gerontology and Geriatrics-Global Ageing Research Network (IAGG-GARN) and the IAGG European Region Clinical Section drew the first recommendations by proposing a complex programme of exercises performed in small groups. Experts recommend the inclusion of stimulating and motivating features, such as music. They also emphasize that detailed guidelines for seniors with impaired mobility are not available [31].

Although previous studies have shown consistent results on the beneficial health effects of exercise, there has been no scientific study on the health-promoting effects of DMT in elderly NH residents in wheelchairs.

The main aim of this research was to assess the influence of physical exercises with DMT elements on the indicators related to strength and fitness of upper limbs and overall functional performance of elderly NH residents in wheelchairs compared to the standard exercise programme and usual care.

We hypothesize that the most significant improvements in strength and fitness of the upper limb and overall functional performance are observed among people who perform physical exercises with elements of DMT.

## Materials and methods

### Trial design

Our study was a randomized controlled trial (RCT) with three groups: group 1, basic exercise (BE); group 2, physical exercise with elements of dance movement therapy (PED); and group 3, usual care (CO). Outcome assessments were performed at baseline, 12 weeks after baseline (immediately after the intervention) and 24 weeks after baseline (12 weeks after the intervention). The study was registered in the Sri Lanka Clinical Trials Registry (Registration Number - SLCTR/2018/014 - Date of Registration 16/05/2018. Accessed on https://slctr.lk/trials/1045).

### Participants

**Fig. 1:**
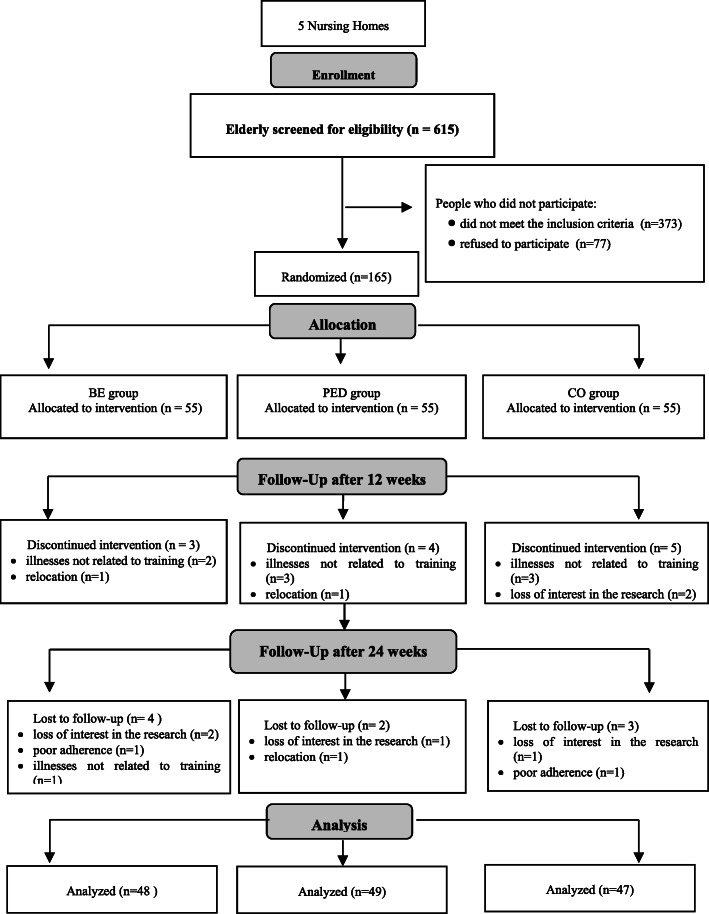
Flow diagram of participants through the study

The study was conducted in five randomly selected NHs for elderly people suffering from chronic physical illnesses in southeastern Poland (the region of the Podkarpackie voivodeship). Participants who were eligible for the trial were required to comply with the following criteria: age, 65–85 years; Mini-Mental State Examination (MMSE) score, ≥ 19 [32]; Geriatric Depression Scale (GDS) score, ≤ 10 points [33]; use of a manual wheelchair as the primary mean of mobility; Berg Balance Scale (BBS) score, 4 < 21 points [34]; Barthel Index (BI) score, 21 < 75 points [35]; and suffered no severe physical disease that could affect participation in the study. The exclusion criteria were: symptoms of cardiovascular diseases, severe systemic diseases, severe circulatory or organic insufficiency, severe neurological disorder and lack of consent to participate in the study. Figure 1, a CONSORT flow diagram, shows the number of participants in particular study arms at each stage of the research.

### Interventions

The intervention for both exercising groups consisted of a 30-min session, two times a week, for 12 weeks in total. Exercises were conducted in subgroups of 6–8 people. The exercise interventions were conducted only in seated position (in wheelchairs). The intensity of effort was moderate on the Borg scale: 11–13 points. Before and after each workout, blood pressure and heart rate were measured and noted.

For the purposes of the exercise programme, 5 qualified physiotherapists were employed. Prior to the intervention, each therapist was instructed in a field of exercise programmes and then randomly assigned to one of the NHs. To eliminate the possibility of differences in programme realization and to ensure the required quality of the intervention, the instructors were periodically interchanged between particular facilities and were under constant supervision of the coordinator. In addition to conducting the exercise programmes, the therapists also monitored and reported any complications that occurred, e.g., pain or injury. Based on their surveillance, the research coordinator could exclude participants from the study in case of any health problem.

The subjects were randomly assigned to one of the three groups:

Group 1, basic exercises (BE): The programme included exercises performed in wheelchairs, including the elements of active, stretching and balance exercises. The exercises were performed according to the following scheme: warm-up (10 min), simple exercises focused on the upper part of the body, such as arm rotation, arm and elbow joint flexing, wrist turning, hand closing and opening, and bending and unbending of the body; main part (15 min), slow active exercises and flexibility and balance exercises; and cool-down (5 min), muscle-relaxing and calming exercises for lowering the heart rate and Stretching exercises for increasing joint mobility of the upper limbs [36].

Group 2, physical exercises with elements of dancing (PED): This programme included exercises performed in wheelchairs. The exercises were performed according to the following scheme: warm-up (5 min), the person conducting the exercises started the session with rhythmic hand clapping to the background music. The participants followed the physiotherapist and simulated activities and phenomena such as driving a car, rustling trees, and collecting fruits, to prepare the upper body muscles; main part (20 min), dance-movement exercises. This phase consisted of simple, easy-to-repeat moves performed in a sitting position (in a wheelchair). The physiotherapist demonstrated a sequence of moves without music, and then the group repeated the sequence to carefully selected and prepared music such as the cha-cha, Zumba, the jive, the macarena, and the hula dance. The example sequence may contain the following moves: hand clapping, wrist turning, and arm and body waving. Additionally, gymnastic accessories, such as sticks, dumb bells, balls, and elastic bands (thera-bands), were used. Each exercise programme contained strengthening and balance exercises. The music was changed each week for 6 weeks and then repeated for another 6 weeks. The character and tempo of the music samples used during sessions were matched for perceptive and mobility capabilities of the elderly. At the end of the main part, the participants were asked to perform spontaneous motive improvisation to stimulate imagination, creativity, and self-expression. Interactions between the subjects were to improve communication and social skills; and cool-down (5 min), simple stretching and breathing exercises with a background of peaceful and relaxing music. After physical activities, the therapist initiated a short discussion on feelings and impressions regarding the exercise session [37].

Group 3, usual care (CO): In the control group, the participants were engaged in their regular daily activities and did not receive any therapeutic intervention.

### Outcome measures

Data were collected at baseline and 12 weeks and 24 weeks after the beginning of the intervention. Data regarding age, gender, education, marital status, years in a NH, years of wheelchair dependence, and chronic diseases were taken from the records kept by the care homes and gathered during interviews with the participants by trained research assistants who were blinded to group assignment. Cognitive status and depression were also assessed by the MMSE [32] and GDS scales [33].

#### Main outcome

Grip strength – Hand grip strength (HGS) was tested using a hand dynamometer (JAMAR plus + hydraulic hand dynamometer, Patterson Medical). Following the recommendations of the American Society of Hand Therapists, the test was performed in a sitting position on a chair without armrests, with the person’s feet resting flat on the floor. The elbow joint angle was 90 degrees, and the forearm was in a neutral position. When asked, the participant performed the maximum squeeze of the dynamometer for 6 s. The test was repeated three times. There was a resting period between each squeeze. The mean value of three squeezes was accounted for (in kg) [38].

#### Secondary outcomes

Upper limb movement was measured using a box and block test (BBT). The test is performed using a box with dimensions of 53.7 × 25.4 × 8.5 cm, divided into two compartments by a partition. In one compartment, there were 150 wooden blocks, each having dimensions of 2.5 × 2.5 × 2.5 cm. The box was placed on a table, with the compartment holding the blocks oriented towards the dominant hand. The participant was asked to move, one by one, the maximum number of blocks from one compartment of a box to another, within 60 s. Higher test scores were indicative of better overall manual dexterity [39].

Strength and endurance test of upper limbs (arm curl test, ACT) – the goal was to complete as many curls as possible in 30 s. The participant sat on a chair, holding a weight of 2.27 kg or 3.63 kg for women and men, respectively. The arm on the dominant side should be immovable, and the arm should hold the weight facing towards the body. The score was the total number of completed curls [40]. The patient’s arm was facing down, along the chair back, perpendicular to the floor, with the forearm and hand in an intermediate position. At the assistant’s signal, the subject performed a bend in the elbow with supination of the forearm and returned to the starting position.

The range of motion (ROM) of the shoulder was measured using a dominant arm goniometer based on the standardized measurement procedure in physical therapy. The ROM measurement included shoulder flexion, abduction, and extension of the dominant side. The ROM of joint action was measured three times, and the averaged number was used [41].

Assessment of upper body flexibility (back scratch test, BS): this test required the participant to bring together the fingers of both hands behind their back – with one arm placed over the upper body and the other arm behind the lower back**.** The distance between the fingertips of both hands was measured. The score given in centimetres (if the fingers overlapped, a positive score was recorded; if the fingertips did not touch, a negative score was recorded) was the indicator of the test. Assessment of lower body flexibility (chair sit-and-reach, CSR): the test required the participant sitting on the edge of a chair to reach forward toward the toes by bending at the hip. The distance is measured between the tip of the fingertips and the toes [42].

Lung capacity was measured based on peak expiratory flow (PEF) and forced expiratory volume in 1 s (FEV1) using a peak flow meter (Peak Flow Metre Microlife PF 100, measuring range: 50–900 l/min and FEV1–0,01–9,99 l). Participants took a deep breath and blew air into the meter as fast as they could. For PEF and FEV1, at least three measurements were performed and the best values were selected [43].

Daily functioning was assessed using the Barthel Index (BI). This tool determines the level of independence in terms of self-sufficiency, mobility, personal hygiene and control over sphincters. The maximum score is 100 points. The interpretation of the individual scores follows the description of the ranges they fit in: 91–100, independent; 62–85, dependent; 21–61, almost completely dependent; and 0–20, completely dependent [44]. To assess the level of independence of older adults, the Katz Index of Independence in Activities of Daily Living (ADL) was used. A score of 6 indicates full functioning, 4 indicates moderate impairment, and 2 or less indicates severe functional impairment [45].

The Berg Balance Scale (BBS) was used to examine elderly people’s static and dynamic balance. Participants were asked to perform 14 types of predetermined movements in a standing and sitting position. A maximum score of 56 points is possible. A score below 20 points indicates a high risk of falling and the need to use a wheelchair by the participants [34].

### Sample size

The sample size was estimated from an a priori power analysis to detect the statistically significant effects of exercise [46]. The sample size was chosen according to the Cohen method, using standard assumptions: 0.05 for significance level, 0.8 for power of test, and 0.5 for effect size, accounting for, according to Cohen, a medium effect size [47]. Based on hand grip strength 37 participants per group were required. To compensate for a possible dropout rate of 20%, our recruitment target per group was 45 NH residents. As more residents wanted to take part in the programme, we decided to include more participants than required.

### Randomization

A central computerized randomization procedure was used to randomly allocate a total of 165 participants: 55 in the basic exercise group (BE), 55 in physical exercises with elements of dancing group (PED), and 55 in the control group (CO). The randomization sequence was generated in blocks, and intervention was allocated by a supervisor researcher who was not involved in the enrolment, intervention, or assessment.

### Statistical methods

Descriptive statistics were used to describe and compare sociodemographic data of participants between groups. Descriptive characteristics are presented as the mean and standard deviation or as a number and percent when appropriate. The comparison of the values of qualitative variables in the groups was performed using the chi-square test. One-way ANOVA was used to assess the differences between groups at the time points post and follow-up. When a significant *p*-value was obtained, Bonferroni post hoc procedures were used to evaluate pairwise differences. The mean difference between treatment groups and the confidence intervals for quantitative variables were also determined. Analyses were conducted at a 0.05 level of significance. A standard per-protocol analysis was performed for each outcome. The analysis was conducted using SPSS Pack 25.0 together with the Exact test module.

## Results

### Demographic and baseline scores

Six hundred and fifteen participants were screened for eligibility, of which 165 were included. Three hundred and seventy-three individuals did not meet the inclusion criteria, and ninety-two refused to participate. The most common reasons for declining to participate were illnesses, reluctance to attend appointments twice a week, and personal reasons interfering with commitment.

Seven participants in the BE group, six in the PED group and eight in the control group (CO group) did not complete the study for the following reasons: illnesses not related to training (BE: *n* = 3; PED: *n* = 3; CO: *n* = 3), loss of interest in the research (BE: *n* = 2; PED: *n* = 1; CO: *n* = 3), poor adherence (BE: *n* = 1; CO: *n* = 1), death (CO: *n* = 1), and relocation (BE: *n* = 1; PED: *n* = 2). Dropout rates for each group were as follows: BE, 12.7%; PED, 10.9%; and CO, 14.6%. The classification of the study participants can be seen in a flow diagram (Fig. 1).

Out of 165 randomized participants, 144 (87.3%) completed the 24-week assessment. The distribution of the surveyed participants across groups was as follows: BE group, 47 participants; PED group, 48 participants; and CO group, 47 participants.

Prior to the start of the exercise programmes, all the tested groups were comparable to one another in terms of cognitive state, functional performance including grip strength, manual dexterity, upper and lower limb endurance, upper and lower body flexibility, range of shoulder mobility, lung capacity, balance and sociodemographic characteristics, except for education level and musculoskeletal diseases. The subjects from the PED group showed a higher level of education than the BE and CO groups. The percentage of people with musculoskeletal diseases was higher in the CO group than in the other groups (Table 1).

**Table 1:** Sociodemographic and clinical characteristics of the participants

Variable			BE group (***n*** = 55)	PED group (***n*** = 55)	CO group (***n*** = 55)	
			Number (%) Mean (SD)			***p*** - value
**Sociodemographic and clinical**						
**Sex**	Female		30 (54.55)	36 (65.45)	32 (58.18)	0.495^c^
	Male		25 (45.45)	19 (34.55)	23 (41.82)	
**Marital status**	Married		9 (16.36)	2 (3.64)	6 (10.91)	0.249^c^
	Widow / widower		25 (45.45)	28 (50.91)	20 (36.36)	
	Divorced		9 (16.36)	10 (18.18)	9 (16.36)	
	Single		12 (21.82)	15 (27.27)	20 (36.36)	
**Education**	Basic		18 (32.73)	8 (14.55)	24 (43.64)	0.020^c^*
	Vocational		33 (60.00)	40 (72.73)	26 (47.27)	
	Higher		4 (7.27)	7 (12.73)	5 (9.09)	
**Length of NH residency**	< 12 moths		6 (10.91)	6 (10.91)	2 (3.64)	0.319^c^
	1–5 years		15 (27.27)	16 (29.09)	14 (25.45)	
	6–10 years		27 (49.09)	26 (47.27)	24 (43.64)	
	over 10 years		7 (12.73)	7 (12.73)	15 (27.27)	
**Length of wheelchair dependence**	< 12 months		1 (1.82)	1 (1.82)	1 (1.82)	0.332^c^
	1–5 years		27 (49.09)	27 (49.09)	22 (40.00)	
	6–10 years		25 (45.45)	24 (43.64)	23 (41.82)	
	over 10 years		2 (3.64)	3 (5.45)	9 (16.36)	
**Chronic disease**	Cardiovascular		39 (70.91)	41 (74.55)	37 (67.27)	0.703^c^
	Musculoskeletal		20 (36.36)	31 (56.36)	32 (58.18)	0.040^c^*
	Neurological		39 (70.91)	39 (70.91)	35 (63.64)	0.638^c^
	Pulmonary		15 (27.27)	7 (12.73)	9 (16.36)	0.127^c^
	Urinary system		16 (29.09)	10 (18.18)	16 (29.09)	0.317^c^
	Otological		19 (34.55)	15 (27.27)	18 (32.73)	0.694^c^
	Ophtamological		20 (36.36)	16 (29.09)	22 (40.00)	0.475^c^
	Digestive system		8 (14.55)	10 (18.18)	7 (12.73)	0.719^c^
**GDS**		5.49 (2.60)	5.58 (2.68)	5.44 (2.69)	5.45 (2.46)	0.951^b^
	No depression 0–5		24 (43.64)	22 (40.00)	29 (52.73)	0.385^c^
	Moderate depression 6–10		31 (56.36)	33 (60.00)	26 (47.27)	
**MMSE**		24.94 (3.37)	24.67 (3.40)	25.13 (3.38)	25.02 (3.36)	0.763^b^
	Normal cognition 30–27		18 (32.73)	22 (40.00)	19 (34.55)	0.723^c^
	Cognitive impairment without dementia 26–24		15 (27.27)	12 (21.82)	18 (32.73)	
	Mild cognitive impairment 23–19		22 (40.00)	21 (38.18)	18 (32.73)	
**Dominant limb**	Right		47 (85.45)	49 (89.09)	42 (76.36)	0.178^c^
	Left		8 (14.55)	6 (10.91)	13 (23.64)	
	Age [years]	74.35 (7.33)	74.40 (8.09)	74.51 (7.37)	74.13 (6.59)	0.962^b^
	Body mass [kg]	161.62 (10.13)	73.61 (14.21)	70.76 (14.06)	68.40 (16.27)	0.188^b^
	Height [cm]	70.92 (14.94)	161.80 (10.91)	161.69 (9.58)	161.38 (10.03)	0.975^b^
	BMI [kg/m^2^]	27.02 (4.44)	28.08 (4.38)	26.92 (3.99)	26.07 (4.76)	0.057^b^
**Main Outcome**						
**Muscle strength**	HGS _R_ [kg]	12.79 (5.43)	12.67 (5.38)	12.74 (5.69)	12.97 (5.30)	0.955^b^
	HGS _L_ [kg]	11.22 (4.52)	11.14 (4.31)	11.09 (4.53)	11.43 (4.79)	0.916^b^
**Secondary Outcomes**						
**Manual dexterity and**	BBT [x]	27.42 (9.96)	27.40 (9.55)	27.42 (9.42)	27.45 (11.02)	> 0.999^b^
**endurance of the upper limb**	ACT [x]	9.17 (3.68)	9.11 (3.80)	9.11 (4.01)	9.29 (3.24)	0.957^b^
**Joint mobility**	Shoulder flexion [degree]	130.48 (20.10)	130.18 (19.34)	131.55 (23.31)	129.73 (17.54)	0.886^b^
	Shoulder extension [degree]	44.45 (17.29)	43.53 (26.76)	44.91 (9.35)	44.91 (10.16)	0.891^b^
	Shoulder abduction [degree]	126.94 (18.96)	125.49 (18.45)	126.96 (20.23)	128.36 (18.39)	0.732^b^
**Flexibility Assessment**	BS _R_ [cm]	−37.74 (22.36)	−37.58 (20.23)	− 37.02 (24.48)	− 38.62 (22.55)	0.931^b^
	BS _L_ [cm]	−39.30 (22.96)	− 39.27 (21.72)	− 39.25 (24.77)	−39.38 (22.72)	> 0.999^b^
	CSR _R_ [cm]	−12.33 (18.10)	−12.35 (19.88)	−12.47 (13.93)	− 12.18 (20.16)	0.996^b^
	CSR _L_ [cm]	−12.45 (17.52)	−12.42 (17.27)	− 12.47 (13.49)	− 12.45 (21.30)	> 0.999^b^
**Lung capacity**	PEF [l/min]	149.28 (42.97)	151.22 (46.59)	147.05 (43.39)	149.56 (39.29)	0.879^b^
	FEV1 [l]	1.17 (0.44)	1.10 (0.45)	1.14 (0.43)	1.27 (0.43)	0.105^b^
**Functional Assessment**	Katz ADL	4.26 (1.10)	4.24 (1.11)	4.27 (1.11)	4.27 (1.09)	0.980^b^
	BI	54.18 (11.56)	54.09 (12.73)	54.36 (12.06)	54.09 (10.23)	0.990^b^
**Body Balance Assessment**	BBS	12.41 (5.60)	12.38 (6.34)	12.67 (5.14)	12.18 (5.33)	0.900^b^

*BE* basic exercises group; *PED* physical exercises with elements of dancing group; *CO* control group; *, statistical significance; ^c,^ chi-square test; ^b^, One-way ANOVA, *SD* standard deviation; *GDS* Geriatric Depression Scale; *MMSE* Mini-Mental State Examination; *BMI* body mass index; *HGS*_*R*_ right-hand grip strength; *HGS*_*L*_ left-hand grip strength, *BBT* box and blocks test; *ACT* arm curl test; *BS*_*R*_ back scratch right arm over; *BS*_*L*_ back scratch left arm over; *CSR*_*R*_ chair sit-and-reach for right leg; *CSR*_*L*_, chair sit-and-reach for left leg; *PEF* Peak expiratory flow; *FEV1* forced expiratory volume in 1 s; *ADL* Activities of Daily Living; *BI* Barthel Index; *BBS* Berg Balance Scale;

The average age of the participants was 74.35 years (SD = 7.33), and no statistically significant differences were found between the groups examined with respect to this parameter.

In the study group, 138 people had dominant right upper limb, and 27 people indicated that the left upper limb was dominant.

The mean right-hand grip strength (HGS_R_) score for the total was 12.79 kg (SD = 5.43), and the left-hand grip strength (HGS_L_) was 11.22 kg (SD = 4.52). The BBT test result for the entire test group was 27.42 blocks (SD = 9.96), and the average ACT was 9.17 repetitions (SD = 3.68). On the other hand, the average BBS score for the entire group was 12.41 (SD = 5.60). The demographic data of the participants and the basic parameters are summarized in Table 1.

### Between-group comparisons over timepoints

#### Between-group comparisons at 12 weeks

##### Main outcome

At 12 weeks of exercise and monitoring, the largest statistically significant change in HGS was recorded in the PED group, compared with the CO group (HGS_R_, HGS_L_: *p* < 0.001) and between BE group compared with the CO group (HGS_R_, HGS_L_: *p* < 0.001). In the PED group, HGS significantly improved, in comparison to the BE group (HGS_R_: *p* = 0,01and HGS_L_: *p* < 0.001) (Table 2.).

**Table 2:** Mean difference scores for each group across time and between-group comparisons at 12 weeks and 24 weeks

	Variable	BE group (***n*** = 52)	PED group (***n*** = 51)	CO group (***n*** = 50)	PED vs BE	PED vs CO	BE vs CO	BE group (***n*** = 48)	PED group (***n*** = 49)	CO group (***n*** = 47)	PED vs BE	PED vs CO	BE vs CO
		Baseline—12 weeks Mean change from baseline (95% CI)				After 12 weeks		Baseline—24 weeks Mean change from baseline (95% CI)			After 24 weeks		
**Main Outcome**													
	HGS _R_ [kg]	5.54 (4.89; 6.19)	6.77 (6.04; 7.50)	−0.77 (−1.06; − 0.48)	*p* = 0,01*	*p* < 0.001*	*p* < 0.001*	4.51 (3.84; 5.18)	6.42 (5.72; 7.11)	−1.42 (− 1.80; − 1.04)	*p* < 0.001*	*p* < 0.001*	*p* < 0.001*
	HGS _L_ [kg]	6.60 (6.08; 7.11)	8.05 (7.51; 8.60)	−0.40 (− 0.56; − 0.24)	*p* < 0.001*	*p* < 0.001*	*p* < 0.001*	6.12 (5.64; 6.61)	7.76 (7.22; 8.30)	−0.95 (−1.22; − 0.69)	*p* < 0.001*	*p* < 0.001*	*p* < 0.001*
**Secondary Outcomes**													
**Manual dexterity**	BBT	9.50 (8.85; 10.15)	12.90 (11.62; 14.18)	−1.50 (−2.01; −0.99)	*p* < 0.001*	*p* < 0.001*	*p* < 0.001*	8.88 (8.15; 9.60)	13.00 (11.69; 14.31)	−2.49 (−3.15; −1.82)	*p* < 0.001*	*p* < 0.001*	*p* < 0.001*
**Arm strength and endurance**	ACT	2.56 (1.98; 3.13)	3.71 (3.13; 4.28)	−0.52 (−0.86; 0.18)	*p* < 0.001*	*p* < 0.001*	*p* < 0.001*	2.40 (1.78; 3.01)	3.67 (3.12; 4.22)	−0.83 (−1.24; − 0.42)	*p* < 0.001*	*p* < 0.001*	*p* < 0.001*
**Joint mobility**	Shoulder flexion [degree]	10.38 (7.58; 13.19)	10.88 (7.91; 13.85)	−1.80 (−2.94; −0.66)	*p* > 0.999	*p* < 0.001*	*p* < 0.001*	5.52 (−0.03; 11.07)	9.69 (6.64; 12.75)	−3.83 (−5.40; −2.26)	*p* = 0,35	*p* < 0.001*	*p* < 0.001*
	Shoulder extension [degree]	4.92 (−2.95; 12.80)	9.71 (8.01; 11.40)	−2.30 (−3.26; −1.34)	*p* = 0,46	*p* < 0.001*	*p* = 0,10	3.67 (−5.02; 12.35)	9.49 (7.75; 11.23)	−2.21 (−3.57; −0.86)	*p* = 0,33	*p* < 0.001*	*p* = 0,33
	Shoulder abduction [degree]	11.87 (8.85; 14.89)	14.94 (12.19; 17.69)	−3.66 (−4.80; −2.52)	*p* = 0,23	*p* < 0.001*	*p* < 0.001*	11.42 (8.23; 14.61)	14.67 (11.93; 17.42)	−4.02 (−5.23; −2.81)	*p* = 0,21	*p* < 0.001*	*p* < 0.001*
**Lung capacity**	PEF	39.83 (32.10; 47.56)	56.10 (41.99; 70.21)	−21.62 (− 28.59; −14.65)	*p* = 0,07	*p* < 0.001*	*p* < 0.001*	9.98 (2.90; 17.06)	41.14 (29.23; 53.06)	−29.23 (−37.40;- 21.07)	*p* < 0.001*	*p* < 0.001*	*p* < 0.001*
	FEV1	0.46 (0.36; 0.56)	0.71 (0.59; 0.84)	−0.15 (−0.20; −0.11)	*p* < 0.001*	*p* < 0.001*	*p* < 0.001*	0.23 (0.15; 0.32)	4.15 (−3.44; 11.74)	−0.36 (− 0.44; − 0.28)	–	–	–
**Flexibility Assessment**	BS _R_ [cm]	8.13 (7.01; 9.26)	14.82 (11.84; 17.81)	−1.20 (−2.23; − 0.17)	*p* < 0.001*	*p* < 0.001*	*p* < 0.001*	6.81 (5.79; 7.84)	13.51 (10.82; 16.20)	−1.79 (−2.96; −0.62)	*p* < 0.001*	*p* < 0.001*	*p* < 0.001*
	BS _L_ [cm]	9.38 (7.71; 1.06)	14.57 (11.38; 17.76)	−1.66 (−2.65; −0.67)	*p* < 0.001*	*p* < 0.001*	*p* < 0.001*	8.23 (6.63; 9.83)	14.00 (10.64; 7.36)	−1.34 (− 1.75; −0.93)	*p* < 0.001*	*p* < 0.001*	*p* < 0.001*
	CSR _R_ [cm]	7.90 (5.37; 10.43)	12.98 (10.76; 15.20)	−1.12 (− 1.59; −0.65)	*p* < 0.001*	*p* < 0.001*	*p* < 0.001*	7.63 (4.94; 10.31)	12.82 (10.55; 15.08)	−2.17 (−2.72; −1.62)	*p* < 0.001*	*p* < 0.001*	*p* < 0.001*
	CSR _L_ [cm]	7.17 (5.70; 8.64)	11.92 (9.93; 13.91)	−0.62 (−1.35; 0.11)	*p* < 0.001*	*p* < 0.001*	*p* < 0.001*	6.58 (5.17; 7.99)	11.78 (9.79; 13.76)	−1.77 (−2.59; −0.94)	*p* < 0.001*	*p* < 0.001*	*p* < 0.001*
**Functional Assessment**	Katz ADL	0.04 (−0.10; 0.17)	0.18 (0.03; 0.32)	0.00 (−0.14; 0.14)	–	–	–	−0.10 (− 0.29; 0.08)	0.12 (− 0.04; 0.28)	−0.68 (− 0.89;-0.47)	*p* = 0,25	*p* < 0.001*	*p* < 0.001*
	BI	0.58 (−0.23; .39)	3.92 (2.53; 5.31)	−1.40 (−2.59; − 0.21)	*p* < 0.001*	*p* < 0.001*	*p* = 0,05*	−0.42 (− 1.25; 0.42)	2.96 (1.44; 4.48)	−4.26 (−5.93; − 2.58)	*p* < 0.001*	*p* < 0.001*	*p* < 0.001*
**Body Balance Assessment**	BBS	1.90 (1.51; 2.30)	2.59 (2.20; 2.98)	−0.40 (− 0.66; − 0.14)	*p* = 0,02*	*p* < 0.001*	*p* < 0.001*	1.88 (1.47; 2.28)	2.39 (1.99; 2.78)	−0.66 (− 0.95; − 0.37)	*p* = 0,15	*p* < 0.001*	*p* < 0.001*

*BE* basic exercises group; *PED* physical exercises with elements of dancing group; *CO* control group; *CI* confidence interval,; *, statistically significant result (Bonferroni post hoc procedures); *HGS*_*R*_ right-hand grip strength; *HGS*_*L*_ left-hand grip strength, *BBT* box and blocks test; *ACT* arm curl test; *BS*_*R*_ back scratch right arm over; *BS*_*L*_ back scratch left arm over; *CSR*_*R*_ chair sit-and-reach for right leg; *CSR*_*L*_ chair sit-and-reach for left leg; *PEF* Peak expiratory flow; *FEV1* forced expiratory volume in 1 s; *ADL* Activities of Daily Living; *BI* Barthel Index; *BBS* Berg Balance Scale. One-way ANOVA was used to assess the differences between groups at the time points post and follow-up

##### Secondary outcomes

Analysis of data collected after 12 weeks revealed that the PED group presented higher statistically significant scores in BBT, ACT, BS_R_, BS_L_, CSR_R_, CSR_L_, BI: *p* < 0.001 and BBS: *p* = 0,02 compared to the BE group. No statistically significant differences regarding the time span in question were noted for joint mobility and PEF, when comparing the PED vs. BE groups. In the PED group BBT, ACT, shoulder flexion, extension, abduction, PEF, FEV1, BS, CSR, ADL, BI, BBS, significantly improved (*p* < 0.001), in comparison to the CO group.

Accordingly, there was a statistically significant difference between the BE and CO groups in the following variables: BBT, ACT, shoulder flexion and abduction, PEF, FEV1, BS_R_, BS_L_, CSR_R_, CSR_L_, BBS: *p* < 0.001 and BI: *p* = 0,05 (Table 2).

#### Between-group comparisons at 24 weeks

##### Main outcome

After 24 weeks of commencing exercises the greatest effects were noted in the PED group, in comparison to both: the BE group and the CO group, in HGS_R_, HGS_L_ (*p* < 0.001) (Table 2.).

##### Secondary outcomes

After 24 weeks from the beginning of the intervention, the most pronounced effects, in relation to all the parameters covered by the study, were found in the PED group compared to the CO group (BBT, ACT, shoulder flexion, extension, abduction, PEF, BS_R_, BS_L_, CSR_R_, CSR_L_, ADL, BI, BBS: *p* < 0.001).

The comparison of the PED group and BE group showed statistically significant differences in almost all areas: BBT, ACT, PEF, BS_R_, BS_L_, CSR_R_, CSR_L_, BI: *p* < 0.001, in favour of the PED group. Only the parameters of shoulder flexion (*p* = 0,35), shoulder extension (*p* = 0,33) and shoulder abduction (*p* = 0,21), ADL (*p* = 0,25) and BBS (*p* = 0,15) revealed no statistically significant differences.

The distinctions related to the said parameters between the BE and CO groups proved to be statistically significant, except for shoulder extension (*p* = 0,33).

The exact data related to the distinctions between the groups in question, as registered 24 weeks after the start of the study, are included in Table 2.

### Mean difference scores for each group across time

The analysis of the impact of the 12-week exercise programme showed the greatest effects on HGS, BBT, shoulder extension and abduction, flexibility, and lung capacity in the participants belonging to the PED group compared to the BE group. In the CO group, on the other hand, a decrease in the values of all the parameters examined was observed. These changes were maintained at the 24 week re-assessment. The average results of the differences for each group after 12 and 24 weeks are shown in Table 2.

#### Main outcome

After 12 weeks of the exercise programme in the PED group, the HGS_R_ and HGS_L_ parameters increased correspondingly by 6.77 kg (95% CI = 6.04–7.50) and 8.05 kg (95% CI = 7.51–8.60), respectively, while in the BE group, they increased by 5.54 kg (95% CI = 4.89–6.19) and 6.60 kg (95% CI = 6.08–7.11), respectively. In the CO group, the HGS_R_ and HGS_L_ decreased by 0.77 kg (95% CI = (− 1.06) – (− 0.48)) and 0.40 kg (95% CI = (− 0.56) – (− 0.24)) respectively. The obtained changes were maintained in both HGS parameters until 24 weeks from the beginning of the intervention (Table 2.).

#### Secondary outcomes

The PED group, after 12 weeks of intervention, showed the following increase in the average values of the parameters: BBT score by 12.90 (95% CI = 11.62–14.18), shoulder flexion by 10.88 deg (95% CI = 7.91–13.85), shoulder abduction by 14.49 deg (95% CI = 12.19–17.69), shoulder extension by 9.71 deg (95% CI = 8.01–11.40), PEF by 56.10 l/min (95% CI = 41.99–70.21), back scratch left arm over (BS_L_) by 14.57 cm (95% CI = 11.38–17.76), back scratch right arm over (BS_R_) by 14.82 cm (95% CI = 11.84–17.81), chair sit-and-reach for left leg (CSR_L_) by 11.92 cm (95% CI = 9.93–13.91) and chair sit-and-reach for right leg (CSR_R_) by 12.98 cm (95% CI = 10.76–15.20). The obtained changes were maintained for most of the aforementioned parameters until 24 weeks after the exercises began (Table 2.).

Regarding the BE group, after 12 weeks of exercises, an increase was noticed in the average values of the following parameters: BBT score by 9.50 (95% CI = 8.85–10.15), shoulder flexion by 10.38 deg (95% CI = 7.58–13.19) shoulder abduction by 11.87 deg (95% CI = 8.85–14.89), shoulder extension by 4.92 deg (95% CI = (− 2.95) – 2.80), PEF by 39.83 l/min (95% CI = 32.10–47.56), BS_L_ by 9.38 cm (95% CI = 7.71–1.06), BS_R_ by 8.13 cm (95% CI = 7.01–9.26), CSR_L_ 7.17 cm (95% CI = 5.70–8.64) and CSR_R_ by 7.90 cm (95% CI = 5.37–10.43). After 24 weeks from the beginning, this trend was maintained in the most variables (Table 2.).

In the CO group, a decrease in values of particular parameters was shown after 12 weeks. The exceptions here were the activities of daily living. After 24 weeks, a further decrease in the parameters tested was observed (Table 2.).

## Discussion

In the literature on this subject, there are only a few reports on physical exercise programmes for wheelchair-bound persons. Given the importance of the issue, it seems to be necessary to develop new preventive programmes, providing complex assistance for persons with impaired mobility. To the best of our knowledge, the study presented in the foregoing is the first to assess the impact of DMT on seniors in wheelchairs living in NHs.

The results of the study demonstrated that persons who participated in 2 exercise sessions per week for a 12-week period had increased parameters that helped assess upper extremity fitness and overall functional performance. The largest positive changes were established in the PED group. Additionally, we observed that the lack of physical exercises in seniors in wheelchairs contributes to decreased manual dexterity of the upper extremities and functional performance.

This study shows that preventive measures, in the form of interventions intended for persons with impaired mobility, are possible and useful for the improvement of the health condition of elderly NH residents. Based on previous studies and recommendations regarding physical activities for seniors, Cordes et al. prepared a programme of cognitive and movement exercises. The research aim was to determine the limits of effectiveness and feasibility for the intervention comprising complex physical exercises for weak, elderly NH residents in Germany [24]. A taskforce, under the auspices of IAGG-GARN recognizing the specificity of the long-term care system, developed recommendations for exercise programmes for seniors under such care. To promote the implementation of the proposed recommendations in the real life of NHs, it is crucial to take into account residents’ desires, preferences, beliefs, and attitudes toward physical activities. To confirm that our recommendations in terms of overall physical activity and exercise training are appropriate and effective for institutionalized older adults, further studies are needed [31].

The results of our study may be useful when developing further strategies that aim to improve or maintain the health of NH senior residents.

Our study compared the effectiveness of two programmes of group exercises: one group taking basic exercises (BEs) and the other group performing physical exercises with elements of dancing (PED). Related studies by other authors emphasize the positive impact of regular physical exercises on the functional performance of seniors who do not have any disabilities [40, 48]. On the other hand, there is no clear and coherent evidence for the beneficial influence of physical exercises on the fitness of elderly wheelchair users living in NHs. Additionally, in their systemic review, Weening-Dijksterhuis et al. proposed that an exercise programme for seniors under institutional care should be a combination of progressive strength training, body balance and functional training. The recommended intensity starts with moderate to high, three times a week, lasting at least for 10 weeks [49]. Many authors present programmes of physical exercises taken in sitting positions that are commonly employed in clinical practice [48]. Multiple morbidities, fear of falling and advanced age may impede elderly persons from participating in organized exercises of this kind [50]. Exercises performed in sitting positions can be utilized both by independent elderly persons and weak seniors, as such exercises are relatively safe [51]. In their study of chair-based exercises aimed at examining the acceptability, feasibility and tolerability of intervention, Robinson et al. demonstrated great interest of care centres as well as seniors taking part in the study [50]. In their systematic review of chair-based exercise programmes, Anthony et al. show that physical exercises of this kind are employed as an alternative for weaker seniors, but their health benefits are uncertain. The authors emphasize the need for further, well-designed studies [18]. The results of our study confirm the effectiveness of exercises taken in a sitting position by wheelchair-bound seniors under institutional care in NHs.

We evidenced that seniors in wheelchairs improved their hand grip strength, upper and lower body flexibility, ROM of shoulder, manual dexterity, ADL and body balance after 12 weeks of physical exercises. The most positive changes were noted in participants from the PED group. Short-term effects lasted for almost 24 weeks after the start of the study. Additionally, the hand grip strength results of the left and right extremities exceeded the suggested minimum clinically significant difference, reaching HGS_R_ = 6.42 and HGS_L_ = 7.76 for the PED group and HGS_R_ = 4.51 and HGS_L_ = 6.12 for the BE group, respectively [52, 53].

The study sample had generally low BBS scores at baseline (average 12.41 points), and after 12 weeks of the intervention, the average scores increased by 1.90 points in the BE group and 2.59 points in the PED group. This is considered a small meaningful change for older people. In their previous studies, Lazowski et al. reported that the Berg balance scores of the sated group (using a wheelchair or requiring assistance with transfers) after 4 months of exercises did not change. The only parameter improved was shoulder strength. Moreover, authors encourage more researchers to accept the challenges, as well as the rewards of working with this frail population [54]. In a prospective, randomized, controlled, semicrossover trial, Baum et al. showed a relatively insignificant impact on the Berg Scale score after a strength and flexibility exercise programme in a frail long-term care facility. According to the authors, this is not surprising because the exercise programme was not focused on balance improvement. Researchers point out that the advantage of such a programme is providing not only recreational activity for the resident but also therapeutic benefits [55].

A decrease in functional performance was observed in elderly persons with impaired mobility who did not perform physical exercises regularly. This correlation was acknowledged in studies carried out by other authors [56, 57]. Maintaining optimal physical fitness by performing physical activity positively influences upper limb fitness, especially manual dexterity. Falconer et al. and Williams et al. demonstrate that manual dexterity is strongly correlated with seniors’ level of independence. The limitation of manual dexterity is accompanied by limitations in terms of ADL, as well as increased dependence on others [58, 59]. Incel et al. found that decreasing manual dexterity and hand grip strength influence dependency on daily functioning and affect seniors’ quality of life [60]. The study of the authors shows that even wheelchair-bound seniors can improve their functional performance by taking physical exercises with elements of DMT [60]. Cross-sectional studies have shown that seniors who perform dance evidence greater flexibility, postural stability, body balance, physical response time and cognitive ability [61]. Other authors emphasize that dance, regardless of its style, can significantly improve strength, endurance and other aspects of functional performance in elderly persons [62, 63]. Unlike traditional physical exercises, DMT has the potential to become a universal activity that can be adjusted to age and physical limitations, and they can contribute to the improvement of mental health and social interactions [64].

The results of this study indicate that DMT highly influences the improvement of upper extremity fitness and overall functional performance. In turn, a lack of physical stimulation causes health problems and deteriorates functional performance, which has a negative impact on the public health system by increasing the costs of care in NHs [14].

The results of the study presented here demonstrate that engagement of seniors in physical exercises with DMT elements prolongs their independence period by improving manual dexterity in activities of daily living, which is reflected in the improved quality of life of elderly persons and decreased health care costs. Effective and low-cost solutions are essential for the improvement of the functional performance of elderly, wheelchair-bound persons living in NHs. The results of the authors’ study confirm the need for regular rehabilitation to avoid functional and psychosocial disability. A practical aspect of this study is the fact that it indicates simple and low-cost exercise programmes for seniors with impaired mobility.

This study had many strengths but also some limitations. First, due to the inclusion criteria applied, only seniors moving around in manually operated wheelchairs were included, which implied that the weakest residents who use electronic wheelchairs were excluded from the study. Second, the homogeneity of the intervention was limited due to the large number of NHs involved in the study. To counteract that fact, the physiotherapists received numerous trainings and were being interchanged between NHs. Third, the authors did not carry out an assessment after 36 weeks. When designing further studies, the sampling points at the 6- and 12-month marks should be scheduled to assess long-term effects.

## Conclusions

In summary, the 12-week programme of physical exercises, with elements of dance movement therapy, is the most effective for improving hand grip strength, manual dexterity of the upper limb and functional fitness of wheelchair-bound seniors who live in NHs.

Based on this complex programme, with elements of strength, body balance and endurance training, further studies may also be able to conceptualize important action recommendations and guidelines promoting the health of NH residents with impaired mobility.

## Data Availability

All data used in this study were stored at https://repozytorium.ur.edu.pl/handle/item/5911

## Abbreviations

ACT: Arm curl test
ADL: Activities of daily living
BBS: Berg balance scale
BBT: Test box and blocks
BE: Basic exercises
BI: Barthel index
BMI: Body mass index
BS: Back scratch
BS_R_: Back scratch right arm over
BS_L_: Back scratch left arm over
CI: Confidence interval
CO: Control
CSR: Chair sit and reach
CSR_R_: Chair sit-and-reach for right leg
CSR_L_: Chair sit-and-reach for left leg
DMT: Dance movement therapy
FEV1: Forced expiratory volume in 1 s
GDS: Geriatric depression scale
HGS: Hand grip strength
HGS_L_: Left-hand grip strength
HGS_R_: Right-hand grip strength
IAGG-GARN: The international association of gerontology and geriatrics-global aging research network
MMSE: Mini-mental state examination
NH: Nursing home
PED: Physical exercises with elements of dance movement therapy
PEF: Peak expiratory flow
RCT: Randomized controlled trial
ROM: The range of motion
SD: Standard deviation

## References

[1] Statistics Poland. CSO, Demographic Surveys and Labour Market Department; Population projection 2014-2050. Available online: https://stat.gov.pl/en/topics/population/population-projection/population-projection-2014-2050,2,5.html. Accessed 13 May 2020.

[2] United Nations, Department of Economic and Social Affairs, Population Division; World Population Prospects: The 2015 Revision, Vol. I: Comprehensive Tables. Available online: https://population.un.org/wpp/Publications/Files/WPP2015_Volume-I_Comprehensive-Tables.pdf, 10.18356/b793d926-en. Accessed 13 May 2020.

[3] Mortenson WB, Miller WC, Backman CL, Oliffe JL (2011). Predictors of mobility among wheelchair using residents in long-term care. Arch Phys Med Rehabil.

[4] Simmons SF, Schnelle JF, MacRae PG, Ouslander JG (1995). Wheelchairs as mobility restraints: predictors of wheelchair activity in nonambulatory nursing home residents. J Am Geriatr Soc.

[5] Visagie S, Mlambo T, van der Veen J, Nhunzvi C, Tigere D, Scheffler E (2015). Is any wheelchair better than no wheelchair? A Zimbabwean perspective. Afr J Disabil.

[6] McPhee JS, French DP, Jackson D, Nazroo J, Pendleton N, Degens H (2016). Physical activity in older age: perspectives for healthy ageing and frailty. Biogerontology..

[7] Marcos-Pardo PJ, Orquin-Castrillón FJ, Gea-García GM, Menayo-Antúnez R, González-Gálvez N, Vale R, Martínez-Rodríguez A (2019). Effects of a moderate-to-high intensity resistance circuit training on fat mass, functional capacity, muscular strength, and quality of life in elderly: a randomized controlled trial. Sci Rep.

[8] Carment L, Abdellatif A, Lafuente-Lafuente C, Pariel S, Maier MA, Belmin J, Lindberg PG (2018). Manual dexterity and aging: a pilot study disentangling sensorimotor from cognitive decline. Front Neurol.

[9] Chen HY, Wang CY, Lee MY, Tang PF, Chu YH, Suen MW (2010). A hierarchical categorisation of tasks in mobility disability. Disabil Rehabil.

[10] Bendayan R, Cooper R, Wloch EG, Hofer SM, Piccinin AM, Muniz-Terrera G (2017). Hierarchy and speed of loss in physical functioning: a comparison across older U.S. and English men and women. J Gerontol A Biol Sci Med Sci.

[11] Ćwirlej-Sozańska A, Wilmowska-Pietruszyńska A, Sozański B (2018). Wiśniowska-Szurlej, analysis of chronic illnesses and disability in a community-based sample of elderly people in south-eastern Poland. Med Sci Monit.

[12] Best KL, Miller WC (2011). Physical and leisure activity in older community-dwelling canadians who use wheelchairs: a population study. J Aging Res..

[13] Jansen CP, Diegelmann M, Schnabel EL, Wahl HW, Hauer K (2017). Life-space and movement behaviour in nursing home residents: results of a new sensor-based assessment and associated factors. BMC Geriatr.

[14] Ćwirlej-Sozańska A, Wilmowska-Pietruszyńska A, Sozański B (2018). Validation of the polish version of the World Health Organization disability assessment schedule (WHODAS 2.0) in an elderly population (60-70 yearsold). Int J Occup Saf Ergon.

[15] World Health Organization, 2009. Available online: http://www.who.int/. Accessed on 13 May 2020.

[16] Rabbia J (2010). Dance as a community-based exercise in older adults. Top Geriatr Rehabil.

[17] Phillips-Silver J, Trainor LJ (2007). Hearing what the body feels: auditory encoding of rhythmic movement. Cognition..

[18] Anthony K, Robinson K, Logan P, Gordon AL, Harwood RH, Masud T (2013). Chair-based exercises for frail older people: a systematic review. Biomed Res Int.

[19] Cordes T, Schoene D, Kemmler W, Wollesen B. Chair-based exercise interventions for nursing home residents: a systematic review. J Am Med Dir Assoc. 2020;17: S1525–8610(20)30842–2. 10.1016/j.jamda.2020.09.042. 10.1016/j.jamda.2020.09.042 33218912

[20] Karmarkar AM, Dicianno BE, Cooper R (2011). Demographic profile of older adults using wheeled mobility devices. J Aging Res..

[21] Britten L, Addington C, Astill S (2017). Dancing in time: feasibility and acceptability of a contemporary dance programme to modify risk factors for falling in community dwelling older adults. BMC Geriatr.

[22] Ball V, Corr S, Knight J, Lowis MJ (2007). An investigation into the leisure occupations of older adults. Br J Occup Ther.

[23] Warms CA, Whitney JD, Belza B (2008). Measurement and description of physical activity in adult manual wheelchair users. Disabil Health J.

[24] Cordes T, Bischoff LL, Schoene D, Schott N, Voelcker-Rehage C, Meixner C, Appelles LM, Bebenek M, Berwinkel A, Hildebrand C, Jöllenbeck T, Johnen B, Kemmler W, Klotzbier T, Korbus H, Rudisch J, Vogt L, Weigelt M, Wittelsberger R, Zwingmann K, Wollesen B (2019). A multicomponent exercise intervention to improve physical functioning, cognition and psychosocial well-being in elderly nursing home residents: a study protocol of a randomized controlled trial in the PROCARE. BMC Geriatr.

[25] Kuan SC, Chen KM, Wang C (2012). Effectiveness of qigong in promoting the health of wheelchair-bound older adults in long-term care facilities. Biol Res Nurs.

[26] Chen KM, Kuo CC, Chang YH, Huang HT, Cheng YY (2017). Resistance band exercises reduce depression and behavioral problems of wheelchair-bound older adults with dementia: a cluster-randomized controlled trial. J Am Geriatr Soc.

[27] Thurm F, Scharpf A, Liberman N, Kolassa S, Elbert T, Lüchtenberg D (2011). Improvement of cognitive function after physical movement training in institutionalized very frail older adults with dementia. Gero Psych.

[28] Eyigor S, Karapolat H, Durmaz B, Ibisoglu U, Cakir S (2009). A randomized controlled trial of Turkish folklore dance on the physical performance, balance, depression and quality of life in older women. Arch Gerontol Geriatr.

[29] Murrock CJ, Graor CH (2014). Effects of dance on depression, physical function, and disability in underserved adults. J Aging Phys Act.

[30] Chin A, Paw MJ, van Uffelen JG, Riphagen I, van Mechelen W (2008). The functional effects of physical exercise training in frail older people: a systematic review. Sports Med.

[31] De Souto BP, Morley JE, Chodzko-Zajko WH, Pitkala K, Weening-Djiksterhuis E, Rodriguez-Mañas L (2016). Recommendations on physical activity and exercise for older adults living in Long-term care facilities: a taskforce report. J Am Med Dir Assoc.

[32] Fryderyk-Łukasik M (2015). Comprehensive geriatric assessment in everyday geriatric and caring practice. Geriatr Opieka Długoter.

[33] Albiński R, Kleszczewska-Albińska A, Bedyńska S (2018). Geriatric depression scale (GDS). Validity and reliability of different versions of the scale-review. Psychiatr Pol.

[34] Liu T-W, Ng GYF, Ng SSM (2018). Effectiveness of a combination of cognitive behavioral therapy and task-oriented balance training in reducing the fear of falling in patients with chronic stroke: study protocol for a randomized controlled trial. Trials.

[35] Chen KM, Li CH, Huang HT, Cheng YY (2016). Feasible modalities and long-term effects of elastic band exercises in nursing home older adults in wheelchairs: a cluster randomized controlled trial. Int J Nurs Stud.

[36] Chodzko-Zajko WJ, Proctor DN, Fiatarone-Singh M, Minson C, Nigg C, American College of Sports Medicine (2009). American college of sports medicine position stand. exercise and physical activity for older adults. Med Sci Sports Exerc.

[37] Prebola, Madelyn A. Dance Therapy Action Plan: Improving Body Posture and Quality of Life in Older Patients. Senior Honors Theses & Projects. 2014;386. Available online: https://commons.emich.edu/honors/386. Accessed 13 June 2020.

[38] Roberts HC, Denison HJ, Martin HJ, Patel HP, Syddall H, Cooper C, Sayer AA (2011). A review of the measurement of grip strength in clinical and epidemiological studies: towards a standardised approach. Age Ageing.

[39] Platz T, Pinkowski C, van Wijck F, Kim IH, di Bella P, Johnson G (2005). Reliability and validity of arm function assessment with standardized guidelines for the Fugl-Meyer test, action research arm test and box and block test: a multicentre study. Clin Rehabil.

[40] Kieffer HS, Lehman MA, Veacock D, Korkuch L (2012). The effects of a short-term novel aquatic exercise program on functional strength and performance of older adults. Int J Exerc Sci.

[41] Scott O (1965). B. universal goniometer. Rheumatology..

[42] Jones CJ, Rikli RE (2002). Measuring functional fitness of older adults. J Active Aging.

[43] Crittenden CN, Pressman SD, Cohen S, Janicki-Deverts D, Smith BW, Seeman TE (2014). Social integration and pulmonary function in the elderly. Health Psychol.

[44] Fi M, Dw B (1965). Functional evaluation: the Barthel index. Md State Med J.

[45] Katz S, Ford AB, Moskowitz RW, Jackson BA, Jaffe MW, Studies of illness in the aged (1963). The index of Adl: a standardized measure of biological and psychosocial function. Jama..

[46] Faul F, Erdfelder E, Lang AG (2007). Buchner a G*power 3: a flexible statistical power analysis program for the social, behavioral, and biomedical sciences. Behav Res Methods.

[47] Cohen J (1992). A power primer. Psychol Bull.

[48] Sherrington C, Whitney JC, Lord SR, Herbert RD, Cumming RG, Close JC (2008). Effective exercise for the prevention of falls: a systematic review and meta-analysis. J Am Geriatr Soc.

[49] Weening-Dijksterhuis E, de Greef MH, Scherder EJ, Slaets JP, van der Schans CP (2011). Frail institutionalized older persons: a comprehensive review on physical exercise, physical fitness, activities of daily living, and quality-of-life. Am J Phys Med Rehabil.

[50] Robinson KR, Long AL, Leighton P, Armstrong S, Pulikottill-Jacob R, Gladman J (2018). Chair based exercise in community settings: a cluster randomised. BMC Geriatr.

[51] Abe T, Fujii K, Hyodo K, Kitano N, Okura T (2018). Effects of acute exercise in the sitting position on executive function evaluated by the Stroop task in healthy older adults. J Phys Ther Sci.

[52] Lang CE, Edwards DF, Birkenmeier RL, Dromerick AW (2008). Estimating minimal clinically important differences of upper extremity measures early after stroke. Arch Phys Med Rehabil.

[53] Bohannon RW (2019). Minimal clinically important difference for grip strength: a systematic review. J Phys Ther Sci.

[54] Lazowski DA, Ecclestone NA, Myers AM, Paterson DH, Tudor-Locke C, Fitzgerald C, Jones G, Shima N, Cunningham DA (1999). A randomized outcome evaluation of group exercise programs in long-term care institutions. J Gerontol A Biol Sci Med Sci.

[55] Baum EE, Jarjoura D, Polen AE, Faur D, Rutecki G (2003). Effectiveness of a group exercise program in a long-term care facility: a randomized pilot trial. J Am Med Dir Assoc.

[56] Telenius EW, Engedal K, Bergland A (2015). Long-term effects of a 12 weeks high-intensity functional exercise program on physical function and mental health in nursing home residents with dementia: a single blinded randomized controlled trial. BMC Geriatr.

[57] Grönstedt H, Frändin K, Bergland A, Helbostad JL, Granbo R, Puggaard L, Andresen M, Hellström K (2013). Effects of individually tailored physical and daily activities in nursing home residents on activities of daily living, physical performance and physical activity level: a randomized controlled trial. Gerontology..

[58] Falconer J, Hughes SL, Naughton BJ, Singer R, Chang RW, Sinacore JM (1991). Self report and performance-based hand function tests as correlates of dependency in the elderly. J Am Geriatr Soc.

[59] Williams ME, Hadler NM, Earp JL (1982). Manual ability as a marker of dependency in geriatric women. J Chron Dis.

[60] Incel NA, Sezgin M, As I, Cimen OB, Sahin G (2009). The geriatric hand: correlation of hand-muscle function and activity restriction in elderly. Int J Rehabil Res.

[61] Hwang PW, Braun KL (2015). The effectiveness of dance interventions to improve older adults’ health: a systematic literature review. Altern Ther Health Med.

[62] Machacova K, Vankova H, Volicer L, Veleta P, Holmerova I (2017). Dance as prevention of late life functional decline among nursing home residents. J Appl Gerontol.

[63] Kaltsatou AC, EL K, Anifanti MA, Douka SI, Deligiannis AP (2014). functional and psychosocial effects of either a traditional dancing or formal exercising training program in patients with chronic heart failure: a comparative randomized controlled study. Clin Rehab.

[64] Kattenstroth JC, Kalisch T, Kolankowska I, Dinse HR (2011). Balance, sensorimotor, and cognitive performance in long-year expert senior ballroom dancers. J Aging Res.

